# Reducing Food Waste in Buffet Restaurants: A Corporate Management Approach

**DOI:** 10.3390/foods12010162

**Published:** 2022-12-28

**Authors:** Chi-Mei Emily Wu, Chih-Ching Teng

**Affiliations:** Department of Restaurant, Hotel and Institutional Management, College of Human Ecology, Fu Jen Catholic University, New Taipei City 242062, Taiwan

**Keywords:** food waste reduction, food waste management, buffet restaurant, corporate management approach, foodservice industry

## Abstract

Food waste has become a significant issue in the foodservice industry. However, food waste management in buffet restaurants has rarely been investigated. Considering the popularity of buffet restaurants in Taiwan, this study serves as the first attempt to identify a corporate management approach to food waste reduction in Taiwanese buffet restaurants. The study case comprises two buffet restaurants of a large chain restaurant company in Taiwan. This study uses both individual in-depth interviews and a focus group, comprising 15 managers, chefs, and front-line employees. The results identify various strategies to mitigate food waste in buffet restaurants at different stages of operation: establishing a central kitchen, cooperating with qualified suppliers, accurate forecasting of food demand, aesthetic buffet table design, redesigning the service method, continually monitoring food waste, and proactive communication to customers. The 3R (Reduce–Reuse–Recycle) food waste hierarchy is also developed to encourage buffet restaurant practitioners to design appropriate food waste mitigation programs.

## 1. Introduction

The United Nations (2019) reported that one-third of the world’s food is discarded every year before it has been eaten, amounting to 1.3 billion tons and occupying 1.4 billion hectares of land, equivalent to 28% of the world’s agricultural land [1]. This food waste costs USD 750 billion per year in economic losses and environmental costs. In Europe, the hospitality industry generated 12% of food waste, the third largest proportion after household and manufacturing food waste [2]. The hospitality sector is one of the main sources of food waste, accounting for 17% of total food waste, and more than two-thirds of food waste is avoidable [3]. 

The Food and Agriculture Organization (2014) defined food waste as food that is suitable for human consumption but is not actually eaten by humans and is discarded or neglected after the food has been left to spoil or expire [4]. Pirani and Arafat (2016) defined food waste as unwanted leftover food from guest plates and disposed food from preparation and cooking [5]. Other studies contrast originally edible food waste with originally inedible bio-waste, or avoidable food waste with unavoidable food waste [6,7]. Food loss is generated from the preparation stage, while food waste involves the discarding and spoiling of food at the consumption stage [8,9]. Okazaki et al. (2008) stated that food waste emerges in the production, processing, distribution, and consumption stages from the food supply chain process [10]. Researchers generally acknowledge that food waste is produced in the pre-consumption and post-consumption stages. The current study adopts and defines food waste as avoidably wasted food that is suitable for consumption [6].

Previous researchers have focused on identifying and quantifying food waste from the setting of households [11,12] or hospitality enterprises [13]. Many studies on hospitality food waste tend to adopt a quantitative approach to measuring food waste in restaurants or catering services to understand and make suggestions for the reduction in food waste [5,14]. Moreover, most food waste studies have examined hotels, à la carte restaurants, catering, coffee shops, university dining halls, or cruises for the investigation of food waste [15,16,17]. Limited effort has been made to investigate food waste reduction in independent buffet restaurants. The food waste of buffet restaurants encompasses preparation waste, customer plate leftover waste, and buffet table leftover waste. The service design of a buffet restaurant is that customers pay a fixed price and are encouraged to take all they want instead of what they can consume. This system results in more food waste than in other restaurants due to more uneaten food on the plate and on the service counter [16,17]. Buffets have higher food production and consumption rates and consequently more food waste than à la carte restaurants [18]. 

Buffet-style restaurants are widely recognized as the most food-waste-generating service operations due to the large portions, unnecessary menu choices, unpredictable demand, customer and employee behavior, and restaurant culture [19]. With the increasing eat-out experience of customers and competitive restaurant business environment, the success of buffet restaurant operations depends on the comprehensive and innovative strategic planning of managerial approaches. The major causes of restaurant food waste and managerial strategies for its reduction are categorized as follows. 

*Forecast food demand and menu design*. Effective forecasting of food demand can improve customer satisfaction and minimize food waste. Menu design is the most important factor in food waste mitigation [20]. Previous research indicated that half of the restaurants in Nordic countries recognize menu planning as the critical step to reduce food waste. Lack of or poor menu planning and extensive menu choices are drivers of food waste in the food production chain in restaurants and foodservice outlets [20,21]. Extensive menu items are essential in buffet restaurants in order to attract customers. However, excessive menu items and food quantity can cause food waste. Historic data and in-time reservation records can increase the forecast accuracy of the number of customer arrivals, and food demand should be estimated and planned by the sales forecast [22,23]. Food purchasing, preparation, storage, and handling can also be monitored to prevent deterioration of food [24].

*Measure and monitor food waste.* In order to identify the most common plate waste items, separate waste bins are recommended in both preparation areas and service areas [20]. Cautiously monitoring food waste on the customer plate and listening to customer feedback can reduce food waste [25]. Higher unit price menu items such as fish and meat are less likely to become leftovers on the customer’s plate than lower unit price menu items such as starch and vegetables in restaurants in Taiwan [26]. In buffet restaurants, portion size control is even more important to reduce food waste since the service staff has no direct control over portion size [27,28].

*Communicate and engage employees and customers.* Restaurant employees and customers’ awareness of food waste can help avoid increased food waste. Managerial positions and first-line employees of all-inclusive hotels play important roles in reducing food waste. From menu design, purchasing, and preparation in the kitchen to serving and educating guests about food waste, effective communication with employees and customers about the issues of food waste is crucial. Media campaigns and academic awareness programs can also influence customer behavior [20].

Therefore, it is important to identify and develop food waste mitigation strategies for inadequate knowledge in the buffet restaurant setting. 

In the field of hospitality food waste studies, there have been extended examinations in many developed and Western countries [29]. Government initiatives, awareness campaigns, and financial incentives have been found to significantly decrease food waste [30]. However, different social and cultural backgrounds may lead to different policies and strategies toward food waste [31]. There is a need to fill the gap for the lack of hospitality food waste studies in Asian countries. Therefore, the current study aims to investigate food waste in Taiwanese buffet restaurants. Taiwan is located in southeastern Asia, with an area of 36,000 square kilometers and a population of 23 million [32]. In Taiwan, 9.7 million tons of solid waste was accumulated nationally in 2018, including 4.2 million tons of general waste, 4.7 million tons of resource waste, and 594,992 tons of food waste. Food waste accounts for 6% of solid waste on average, while the average daily general waste was 1.13 kg per person in Taiwan [33]. The Ministry of Finance in Taiwan has found that the number of restaurants in Taiwan is growing by 3% to 6% every year, reaching 136,906 restaurant establishments in 2017. With the booming foodservice industry and the increasing frequency of dining out, food waste management in Taiwan’s restaurant industry is essential [34].

The current study investigates the corporate food waste management approaches adopted in buffet restaurants in Taiwan. More specifically, the objectives of the study are two-fold: (1) to identify and evaluate the food waste at different operation stages; (2) to develop corporate management strategies to minimize food waste in buffet restaurants. This study is expected to help hospitality researchers and practitioners better understand food waste issues in buffet restaurants, as well as to provide suggestions to minimize food waste in buffet restaurants.

## 2. Methods 

### 2.1. Participants and Data Collection

The present study adopted and modified the managerial framework developed by Filimonau and De Coteau (2019) to identify a corporate management approach for the reduction of food waste in Taiwanese buffet restaurants, taking a large chain buffet restaurant in Taipei as an example [13]. The investigation of food waste management incorporated three stages of restaurant operation, namely pre-restaurant (pre-consumption), restaurant (on-site preparation), and consumption. This study utilized a qualitative approach by using a case study to investigate the corporate food waste management of buffet restaurants. Two buffet restaurants of a large chain restaurant company in Taiwan were selected as the sample units due to their popularity and market recognition. According to the DailyView (2019), the two selected buffet restaurants ranked #1 and #8 among the best 20 buffet restaurants in Taiwan, based on the research of the KEYPO social media search engine in 2019 [35]. Regarding the capacity, the two restaurants comprised a regular buffet restaurant with 370 seats and a vegetarian buffet restaurant with 368 seats. The average meal price ranged from USD 26 to 37 for the regular buffet restaurant and from USD 16 to 23 for the vegetarian buffet restaurant. The two case study buffet restaurants provided Chinese, Western, and Japanese cuisines.

Individual, semi-structured, in-depth interviews and a focus group were both applied to understand the broader context of food waste generation at different operation stages and to identify employee and management perspectives on food waste reduction in the buffet restaurant operations. Open-ended interview questions were used to encourage interviewees to express their observations and perspectives [36]. The application of a focus group enabled further analysis and verification of the data collected through the interview stage.

Individual interviews were conducted with on-site chefs, store managers, and waitpersons in order to gather data regarding food waste from their perspectives in various employment positions in a buffet restaurant setting. The purposive sampling approach was followed to select potential interviewees and collect data from the two buffet restaurants. The criteria for selecting participants were chefs with over 10 years of working experience in kitchens and managers and waitpersons that have more than 5 years of working experience in the restaurant industry. 

The interview guideline was developed based on the extensive literature reviews of food waste in hospitality studies [5,13,17]. A pilot test interview was conducted with a buffet restaurant manager to ensure the reliability and validity of the interview guideline. A ten-question interview guideline was finalized and applied to both individual interviews and the focus group. After the demographic questions, the participants were asked to identify the major items of food waste, to describe the procedures to handle the food waste, to define the challenges of food waste mitigation, and to suggest strategies of reducing food waste for buffet restaurants.

The individual interviews were undertaken in October 2019 by inviting 10 participants, comprising two restaurant managers, six chefs, and two first-line waitpersons from the two sample restaurants. The interviews ranged from 30 min to 1 hour. The average age of the participants was 36.8 years, most had high school degrees, the average working experience in the hospitality industry was 15.3 years, and the average working length at the current company was five years. 

Additionally, a focus group was formed at the central kitchen of the company headquarters to understand different perspectives and management practices in terms of food waste in restaurant operations. The central kitchen has operated since 2012 and provides over 200 products to their restaurants under nine different brands. To be eligible for the study, the participants had to have at least 10 years of working experience in kitchens or 5 years of experience in administrative management. Eventually, five managers with positions in top management, procurement, food preparation, and safety control agreed to join the focus group discussion. The two-hour focus group discussion was conducted in November 2019, and one of our researchers with 20 years of research experience in the field of green hospitality and sustainable development hosted the focus group discussion. The participants’ aged ranged from 30–60 years, with an average of 16.6 years of working experience in the hospitality industry. The demographic information of all participants is presented in Table 1. 

### 2.2. Data Analysis

All interviews and the focus group discussion were audio-recorded and transcribed into text for data analysis. Thematic analysis was applied to identify common themes or ideas derived from the data collection in the qualitative research. Six steps of thematic analysis were followed, including being familiar with the data, coding the data, developing primary themes, revising the themes, defining the themes, and writing up the final report [37,38]. The data were manually analyzed by two researchers who have experience in qualitative research and knowledge of food waste issues. Both researchers reached a consensus and formulated themes of food waste reduction in buffet restaurants. To test the validity of the study, an independent expert in the field of qualitative research was invited to examine the merged themes by the research team. 

In addition, to collect more data for comparison, the researchers obtained the average amount of food waste of the two studied restaurants. The food waste included the kitchen waste (preparation, cutting, and loss), food waste from the buffet counter, and customer plate waste. The customer waste was separated from edible food waste and inedible food waste when the service staff collected the food waste. The food waste was weighted and recorded by barrels. The total waste amount was weighted and reported by a contracted food waste specialist company. The provided data were cross-validated with the interview results. 

## 3. Results and Discussion 

### 3.1. Food Waste per Customer and Causes in Buffet Restaurants

According to the results of the obtained data from the two studied restaurants, the average food waste was 115 g per customer in the regular buffet restaurant and 112 g per customer in the vegetarian buffet restaurant. More specifically, the average food waste at the regular buffet restaurant was 125 g per customer on weekdays and 92 g per customer on weekends. At the vegetarian buffet restaurant, an average of 133 g of food waste was produced per customer on weekdays and 62.5 g per customer on weekends. Interestingly, the buffet restaurants generated less food waste per customer at weekends than on weekdays, implying that the buffet restaurants needed to provide a variety of food items for guests even during off-peak weekdays and thus produced more food waste per customer on weekdays. Conversely, the studied buffet restaurants generated less food waste on weekends due to more accurate forecasts of numbers of guests and food demand from family and group bookings. 

A previous study examined the food waste in an à la carte Chinese restaurant in the UK [39]. The average food waste per guest was 138 g. The results also indicated that 60% of the food waste was generated on weekends due to customer plate waste. People like to eat out to celebrate their weekends. The kitchens generated the most food waste during weekdays due to poor demand and production forecasting. The finding of the current study is in line with past research which emphasizes the significance of customer forecasting. Accurate forecasting on incoming guests leads to a more accurate food demand and thus reduces unexpected food waste. 

Another interesting result was that the vegetarian buffet restaurant generated significantly less food waste than the regular buffet restaurant on weekends. The chefs and managers of the vegetarian buffet restaurant confirmed that most of their weekend guests were vegetarians and health-conscious people, who typically paid more attention than others to the issue of the environment and thus generated less food waste. The finding may demonstrate the positive impact of environmental and health-conscious vegetarian guests on food waste mitigation. The analytical results may also elucidate the issue of food waste in vegetarian restaurants, which has not been addressed previously in academic research.

*“Most of our food waste is in the stage of preparation food, such as cutting vegetables. However, compared to a regular buffet restaurant, our food waste is much lower, since we do not serve meet products. Meanwhile, most of our guests finish their plates”*.

(interviewee 6)

The studied central kitchen generated 200 kg–400 kg of food waste per day. Most of the food waste at the central kitchen was inedible food, such as peels, bones, and eggshells. A case study of a buffet restaurant in a five-star international hotel in Malaysia found that an average of 1100 g waste per customer for lunch and 1000 g waste per customer for dinner were generated. The buffet food waste in the two current studied restaurants was only one-tenth of the food waste of the findings of Papargyropoulou et al. (2016) [17]. The results of the current study indicate that the central kitchen positively affected food waste mitigation patterns.

*“I think the central kitchen taking the order from the restaurant to produce food is a good way to reduce food waste. The more you can prepare at the central kitchen, the less food waste you will generate at the restaurant outlets”*.

(interviewee 2)

Central kitchens play a significant role in ensuring standardized management and decreasing the food waste of chain buffet restaurants. The functions of the central kitchen in chain buffet restaurants include unified procurement of ingredients, quality control, pre-treatment of raw food material, preparing the ready-to-cook products, reducing the preparation work at the on-site restaurants, and minimizing the volume of food waste.

In addition to the provided data, all interviewees were asked to identify the major categories of food waste from their observations and experience. The interviewees reported that the major contributors of food waste of regular buffet restaurants were dairy, sauces and soups, and cereals (rice and pasta). For the vegetarian buffet restaurant, dairy, cereals, and sauces and soups ranked as the top three food waste categories. However, this study has different findings from Filimonau et al. (2021), which found that rice, noodles, vegetables, meat, and seafood were the most common wasted categories in a Chinese restaurant in the UK [39]. Betz et al. (2015) also found contrarily that starchy accompaniments (potatoes, rice, and pasta) and vegetables accounted for the largest proportions of food waste in Swiss restaurants [22]. 

### 3.2. The Management Approach to the Reduction of Buffet Food Waste

**Figure 1:**
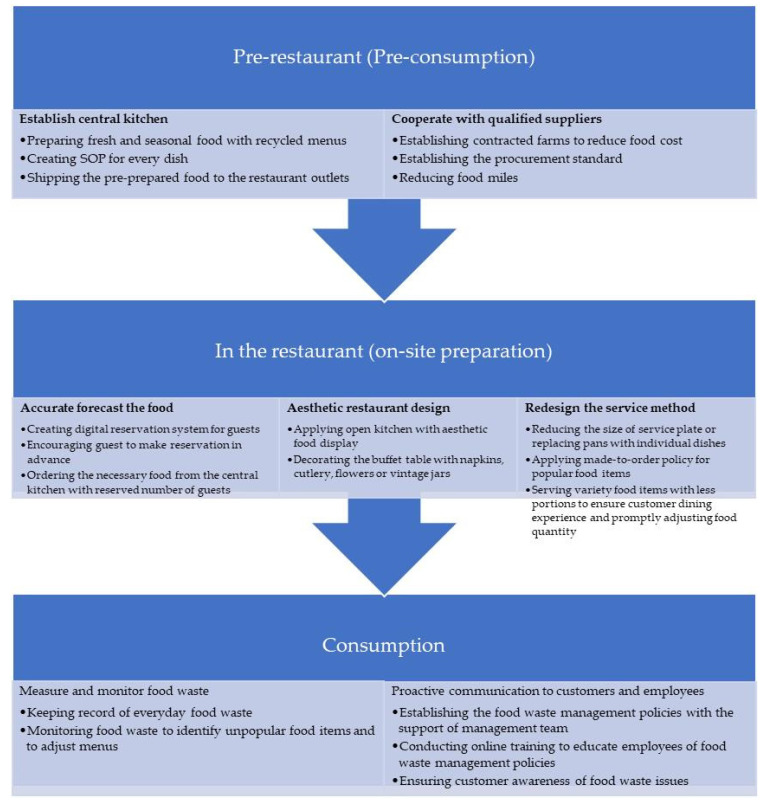
Corporate management approach to the reduction of food waste in buffet restaurant.

As the majority of restaurant food wastage occurs at the stages of food preparation and consumption (WRAP, 2011), understanding where and why buffet food waste is generated is important for setting appropriate strategies based on different phases of restaurant operation [40]. Based on an analysis of the research data from the interviews and focus group, this study identified a corporate management approach for reducing food waste at different stages in the buffet restaurant operations. Figure 1 summarizes the specific management actions for food waste mitigation in the pre-restaurant, restaurant, and consumption stages recommended by the participants. More specifically, establishing a central kitchen, cooperating with qualified suppliers, accurately forecasting, providing an aesthetic restaurant design, redesigning the service method, measuring and monitoring food waste, and proactively communicating with customers and employees were the most mentioned prevention and mitigation management approaches by the respondents. 

According to Figure 1, all interviewees agreed that establishing a central kitchen and cooperating with qualified suppliers are critical strategies at the pre-restaurant (pre-consumption) stage. In the studied central kitchen, most of the food waste produced in the central kitchen was unavoidable food waste, such as trimming, peeling, bones, and packaging. The central kitchen orders fresh food from contracted farms to minimize food miles and then ships the pre-prepared food to the restaurant outlets only two days in advance. The kitchen staff then start to prepare food after receiving the order from the restaurant outlets. A standard procurement and stock management system is implemented. The recycled seasonal menus are carefully planned and examined. Therefore, the central kitchen can guarantee the quality of food, avoid the spoilage of food, and reduce the food waste at the pre-restaurant stage. As one interviewee stated:

*“The central kitchen produces 30 to 35 items per day. We basically wash, cut, marinate, and pre-treat vegetable and meet products, and then ship them to the restaurants. We can deal with the most difficult and time-consuming production. The more we can produce in the central kitchen, the less food waste will yield at the restaurant”*.

(interviewee 11)

Second, at the restaurant (on-site preparation) stage, accurate forecasting of food demand, an aesthetic buffet table design, and service method redesigning are the most mentioned tactics to lower food waste. As more than 70% of guests have reservations, restaurant outlet managers and chefs can easily estimate the demand and place orders to the central kitchen accordingly. The digital reservation system and guest arrival confirmation by calling the guest one day in advance enable managers and chefs to prepare for different scenarios and adjust the quantity of food promptly and effectively. This is in line with past research which considered demand forecasting as one of the most popular approaches for food waste prevention [41]. 

Additionally, many participants stated that the open kitchen design is considered as an effective service design to decrease customer food waste. An open kitchen can “show chefs” cooking in front of guests according to requests to upgrade the customer dining experience and reduce food waste. Instead of using big pans with large quantities of food, utilizing personalized dish plates can not only reduce food waste but also elevate the aesthetic buffet table design. Staff at the service counter and kitchen need to discuss the appropriate quantity of food. A previous study confirmed that reducing the meal size could result in less food waste, and smaller portion sizes had no effect on customer satisfaction [27]. As an interviewee stated:

*“Most of the food was cooked in the open kitchen with the policy of smaller quantity and more patches in order to control the portion and reduce food waste at the service table”*.

(interviewee 1)

Finally, at the consumption stage of a buffet restaurant, monitoring the type and quantity of food wasted during cooking and on the customer’s plate was the most mentioned and essential approach to the reduction of food waste. The “healthy loss” of the studied restaurants was 30 min. If the food has been on the counter and has not been taken by customers for over 30 min, the chefs will replace it with a new dish due to the poor quality of taste. Therefore, monitoring food waste at the service counter and on the customer’s plate can help to prevent food waste. According to research findings, food waste management at the guest service stage helps restaurant operators to catch information about the popularity of items—information which can be adopted to predict guest needs and future demands and to plan buffet menus that avoid food waste generation.

*“We recorded food waste every day in order to accumulate the number for better estimation for the future”.*

(interviewee 7)

Another important approach to the mitigation of food waste is to communicate with customers and employees. The majority of participants believed that customers contribute most of the food waste in the buffet restaurants. A buffet restaurant can adjust the menu and dishes based on guest preferences and perceived food quality. Although customers can take as much as they want in a buffet restaurant, educating customers to take proper portions is important. A previous study found that self-service, moral persuasion in the form of verbal and written formats, and financial discounts were the most effective methods to prevent food waste in buffet restaurants [42].

*“We will approach customers if they leave a lot of untouched food. We need to know the reasons why they don’t like it. We also provide QR codes to customers for immediate feedback”*.

(interviewee 6)

The respondents also stated that employees’ awareness of food waste issues plays an important role in preventing food waste in the buffet restaurants. The studied restaurants provide online training courses on food safety, food waste management policy, and customer relations to employees through a smartphone application. All employees of this restaurant chain are required to participate in the specific courses based on their positions. Reducing food waste requires cooperation among managerial and corporate support, employee actions, and customer understanding.

*“The training courses cover the professional knowledge and competence, food safety and food waste issues. We need to finish 6-chapter courses within a year”*.

(interviewee 12)

The respondents were asked to provide suggestions to reduce food waste for buffet restaurants. Several interviewees indicated that they were aware of the issue of food waste and preferred to donate the leftover food to food banks or charity groups if the company would agree to implement a policy for the donation of leftover food. However, the food waste policies of the sample restaurants require that unconsumed foods on the buffet table should not be reused to serve guests for the next day. Out of food safety concerns, the company policy also forbids the donation of excess foods or leftovers to charity. Leftovers are first used as employee meals, and anything left is disposed as food waste. Since the Taiwanese government does not have regulations to discharge food donors’ liability after donations, most restaurant operators feel reluctant to donate leftover food [29].

*“I found that most of the food waste occurred after dinner. Since company cannot allow employees to bring leftover food home, most of the leftover after dinner will be thrown away. I think it would be nice if we could donate untouched food to food bank or charity groups”*.

(interviewee 1)

The findings of this study provide theoretical implications by designing a corporate management approach framework which helps buffet restaurant practitioners in developing effective strategies to prevent and reduce food waste. The research found that customer behavior plays the most important role in reducing food waste in buffet restaurants. The findings are similar to those of a study of hotel food waste in Florida [21], while other research considers that the preparation and cooking stages contribute most to food waste [43]. Public awareness campaigns, government regulations, or corporate policies can educate irresponsible customers to prevent food waste in all hospitality sectors.

**Figure 2:**
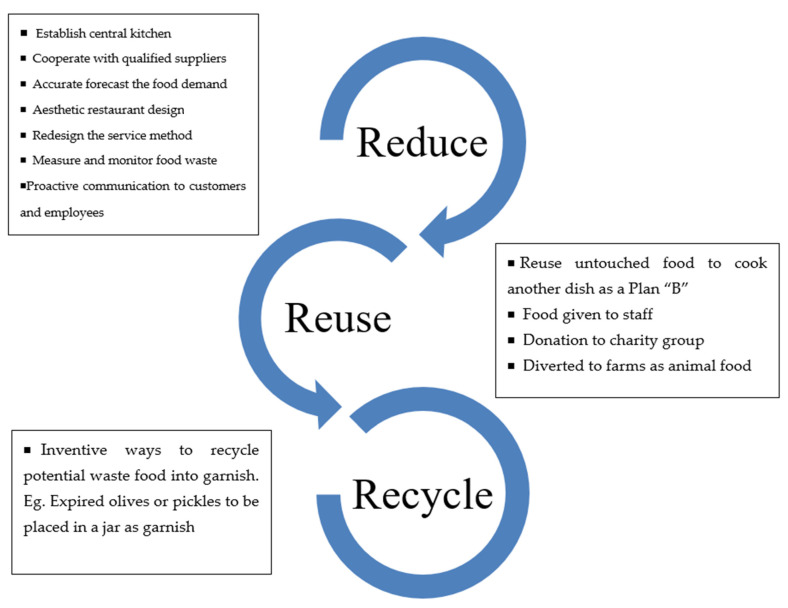
Strategies of hierarchy mitigation food waste in buffet restaurants.

Based on the results of this study, the authors transformed the 3R (Reduce–Reuse–Recycle) framework and developed a hierarchy of food waste mitigation in buffet restaurants, presented in Figure 2. Since the 3R (Reduce–Reuse–Recycle) framework has been utilized successfully in waste management, researchers also applied it on food waste management [44]. In the stage of reducing food waste, establishing a central kitchen and accurately forecasting food demand are considered essential strategies. Meanwhile, reusing leftover food to prepare employee meals or to donate to charity groups and recycling potential waste food into garnishes are strategies suggested by interviewees of the current study. Buffet restaurant managers are encouraged to design a food waste reduction program based on the proposed 3R hierarchy mitigation food waste strategies (see Figure 2).

## 4. Conclusions

Food waste is a significant challenge for the foodservice industry, particularly for buffet restaurants. This study aimed to examine effective management approaches for the reduction of food waste in buffet restaurant operations. The analytical results demonstrate that managers and chefs are aware of food waste and positively respond to the issues. Additionally, several strategies were found to be helpful in decreasing food waste in buffet restaurant operations at different stages of operation, including establishing a central kitchen for restaurant outlets, accurately forecasting the number of meals, proactively communicating with customers, raising awareness of food waste among guests and employees, and donating leftovers to charity organizations. With the increasing number and popularity of buffet restaurants in Taiwan, appropriate management strategies and practices regarding food waste reduction in buffet restaurants are critical to the sustainability of the foodservice industry. This study employed the qualitative method by interviewing on-site practitioners, providing first-hand data and observations of food waste in the buffet restaurant sector. The findings of this study should contribute to the knowledge of food waste management in buffet restaurant operations for both industry practitioners and policy makers.

The practical implication of this study is the development of strategies of hierarchical mitigation of buffet food waste (see Figure 2). Employees of buffet restaurants may recognize food waste interventions and could become appropriate ambassadors to engage with guests to raise public awareness of food waste issues. Corporate policy makers should engage with first-line service staff and chefs to establish effective approaches for food waste disposal. Moreover, strict corporate policies and insufficient government support for food donations are identified as inhibitors of food waste reduction. This result indicates that governmental interventions in policy making and consumer awareness of restaurant food waste can help prevent and reduce food waste in the foodservice sector.

## 5. Limitations and Future Studies

The study has several limitations. The first limitation is the restricted generalization and representation of the results due to it being a case study with a limited number of participants. The results provide an exploratory outlook, not a conclusive analysis. Future studies are encouraged to expand the sample of buffet restaurants in different areas or cultures and collect quantitative data for further food waste analysis in order to better the representation of the results. The second limitation is that interviews and a focus group were the research methods to collect data. However, the risk of social desirability bias sometimes influences the responses of the participants as they tend to answer the interview questions in a “socially acceptable” way [45]. Future studies could collect data from large-scale research of surveys of customers and practitioners. 

## Figures and Tables

**Table 1:** Demographic information of participants.

Code	Gender	Age	Managerial Role	Work Experience in Hospitality Industry	Research Method
1	Male	35	Executive chef	22	Interview
2	Male	49	Head chef	17	Interview
3	Male	43	Head chef	20	Interview
4	Male	40	Store manager	10	Interview
5	Male	34	Team leader of waitpersons	14	Interview
6	Male	35	Executive chef	14	Interview
7	Male	37	Head chef	18	Interview
8	Male	38	Head chef	22	Interview
9	Female	33	Store assistant manager	8	Interview
10	Female	24	Team leader of waitpersons	8	Interview
11	Male	48	Executive chef	25	Focus group
12	Male	31	Food safety manager	6	Focus group
13	Male	41	Procurement manager	23	Focus group
14	Male	42	Head chef	20	Focus group
15	Female	62	Assistant general manager	9	Focus group

## Data Availability

All related data and methods are presented in this paper. Additional inquiries should be addressed to the corresponding author.
